# Prevalence, Risk Factors and Antibiotic Resistance of Extended-Spectrum Beta-Lactamase-Producing *Escherichia coli* in Children Hospitalized with Urinary Tract Infection at King Abdulaziz University Hospital, Jeddah, Saudi Arabia

**DOI:** 10.3390/children11111332

**Published:** 2024-10-31

**Authors:** Abobakr Abdelgalil, Fajr Saeedi, Eilaf Metwalli, Futoon Almutairi, Mayar Felemban, Hadeel Albaradei, Haneen Aseeri, Jawahir Mokhtar, Wesam Baw, Mohamed Sayed

**Affiliations:** 1 Department of Pediatrics, Faculty of Medicine, Cairo University, Cairo 12613, Egypt; mibrahim@kau.edu.sa; 2 Department of Pediatrics, King Abdulaziz University Hospital, Jeddah 21589, Saudi Arabia; eilaf.metwalli@gmail.com; 3 Department of Pediatrics, Faculty of Medicine in Rabigh, King Abdulaziz University, Jeddah 21589, Saudi Arabia; fasaeedi@kau.edu.sa; 4 Faculty of Medicine in Rabigh, King Abdulaziz University, Jeddah 21589, Saudi Arabia; ffaize@stu.kau.edu.sa (F.A.); mfelemban0034@stu.kau.edu.sa (M.F.); hasiri0078@stu.kau.edu.sa (H.A.); 5 Faculty of Medicine, King Abdulaziz University, Jeddah 21589, Saudi Arabia; halbaradei0001@stu.kau.edu.sa; 6 Department of Clinical Microbiology and Immunology, Faculty of Medicine, King Abdulaziz University, Jeddah 21589, Saudi Arabia; jmokhtar@kau.edu.sa; 7 Vaccines and Immunotherapy Unit, King Fahd Medical Research Center, King Abdulaziz University, Jeddah 21589, Saudi Arabia; 8 Al-Noor Specialist Hospital, Makkah 24245, Saudi Arabia; wesambaw@gmail.com

**Keywords:** extended-spectrum beta-lactamase *E. coli*, urinary tract infections, prevalence, risk factors, resistance to antibiotics

## Abstract

Background/Objectives: We aimed to assess the prevalence and risk factors for acquisition of extended-spectrum beta-lactamase (ESBL)-producing *Escherichia coli* (*E. coli*) in children admitted with urinary tract infection (UTI) at a tertiary university hospital in Saudi Arabia, as well as to investigate antibiotic resistance patterns. Methods: This retrospective cross-sectional study involved hospitalized children aged 0–14 years from January 2018 to December 2022 with urine cultures that grew *E. coli* or ESBL-producing *E. coli*. Data of the antimicrobial susceptibility for isolated bacteria were collected. Results: This study analyzed 242 urine samples obtained from 119 children with *E. coli* UTIs. Of these, 20.7% (*n* = 50) were ESBL producers. Previous antibiotic use (last 3 months), prophylactic antibiotic use, prior UTI (last 3 months), recurrent UTIs, and underlying co-morbidities (*p* = 0.011, <0.001, 0.025, <0.001, and 0.013, respectively) had a significant relationship with increased risk of ESBL *E. coli* UTIs. Generally, the highest resistance rates in the ESBL-producing isolates were for ampicillin and third-generation cephalosporin. Conversely, all ESBL-positive isolates were sensitive to meropenem, with variable resistance rates to other antibiotics as amikacin, nitrofurantoin, quinolones and trimethoprim/sulfamethoxazole (2%, 8%, 56% and 64%, respectively). Conclusions: There is a high prevalence of ESBL production among children hospitalized with *E. coli* UTIs. Addressing ESBL UTI risk factors helps to recognize high-risk cases and enhance proper antibiotic use.

## 1. Introduction

Urinary tract infection (UTI) is one of the most prevalent bacterial infections in the pediatric population, occurring in both community and hospital settings. If left untreated, UTI can lead to significant morbidities [1]. *Escherichia coli (E. coli)* is the most frequently identified pathogen encountered among pediatric patients with UTIs, found in 80–90% of cases [2]. 

Antibiotic resistance and the emergence of extended-spectrum beta-lactamase (ESBL) infections are growing concerns in public health. The Center for Disease Control and Prevention (CDC) classified ESBL Gram-negative bacteria as a “serious threat” pathogen in their 2019 report. These bacteria produce beta-lactamase enzymes to resist beta-lactam antibiotics, with the exception of carbapenems and cephamycins. ESBL-producing isolates are becoming more resistant to numerous antibiotic categories such as quinolones and aminoglycosides, which considerably limit treatment options, with empirical therapy typically being ineffective, leading to poor outcomes like septicemia, renal scarring, and prolonged hospitalization [3].

The original dissemination of ESBL-producing UTIs occurred in hospitals, but these infections soon extended to become community-onset UTIs. Some studies have found that recurrent UTIs, previous urological abnormalities, previous antibiotic usage, hospital admissions, presence of a urinary catheter or device, use of suppressive medications, immunocompromised states, chemotherapy, and malignancies are significant risk factors for the acquisition of ESBL UTIs [4].

Globally, ESBL *E. coli* UTIs are increasing. A 2019 CDC report found that between 2012 and 2017, there was a 50% increase in infections attributed to ESBL-producing enterobacteriaceae in the United States. The CDC research showed a considerable increase in hospitalizations for ESBL-producing bacterial infections across all ages, from 141,900 cases and 6300 fatalities in 2012 to 197,400 cases and 9100 deaths in 2017 [5]. Moreover, data from the Study for Monitoring Antimicrobial Resistance Trends (SMART) revealed that the prevalence of UTIs caused by ESBL *E. coli* increased from 7.8 to 18.3% in the United States between 2010 and 2014 [6]. 

There is limited research on the prevalence, risk factors, and antibiotic profiles of ESBL *E. coli* UTIs in hospitalized children, particularly in the Middle East region, where hospitals overuse antibiotics, resulting in antimicrobial resistance to uropathogenic bacteria. Misuse, underuse, or overuse of these antibiotics, combined with disregard for local population susceptibility, invariably led to the upsurge of multi-drug-resistant isolates of these pathogenic bacteria [7].

The current study aimed to elucidate the prevalence, risk factors, and antibiotic resistance of ESBL-producing *E. coli* in urine samples from pediatric patients hospitalized with UTIs.

## 2. Materials and Methods

This retrospective cross-sectional study was carried out at King Abdulaziz University Hospital in Jeddah. Our research followed the ethical norms in the Declaration of Helsinki and was sanctioned by the ethics committee at the hospital (reference number 250-23). Obtaining written consent from patients was not required. This study included hospitalized pediatric patients aged 0–14 years, from January 2018 to December 2022, with urine cultures that grew *E. coli* or ESBL-producing *E. coli* (from a microbiology lab computerized database), confirmed by the presence of significant bacteriuria with a minimum of 100,000 CFU/mL (colony-forming units per milliliter) of a single urinary pathogen collected from a clean-catch specimen for children who had completed toilet training, or a minimum of 50,000 CFU/mL of a single urinary pathogen collected via catheterization according to the clinical practice guidelines of the American Academy of Pediatrics (AAP). UTIs were identified based on an abnormal urine analysis and a positive urine culture following the recommendations set by the AAP [8]. Moreover, codes for discharge diagnosis of these patients were “Urinary tract infection”, “UTI”, “Cystitis”, and/or “Pyelonephritis”. Patients with mixed bacterial growth and those with urine cultures that did not match the AAP criteria for UTIs were excluded. Multiple isolates from the same patient were considered distinct isolates only if they were collected from separate UTI episodes that occurred at least two weeks apart or if they were collected during the same episode but exhibited different antimicrobial susceptibility patterns, indicating distinct strains, thus avoiding overrepresentation of any particular strain.

The patients’ data were collected from our hospital computer system using a standardized checklist. This included demographic information and potential risk factors such as prior antibiotic use within the last three months, prophylactic antibiotic use, recurrent UTIs, previous hospitalization or genitourinary surgery within the last three months, the presence of urinary devices, underlying comorbidities, and genitourinary abnormalities such as vesicoureteral reflux (VUR). 

Clinical manifestations such as fever and urinary symptoms were documented. Data on the UTI-causing organism, whether *E. coli* or ESBL *E. coli*, were incorporated. The laboratory tests performed comprised complete blood count (CBC), C-reactive protein (CRP), blood urea nitrogen (BUN), creatinine, urine analysis, and culture with antibiotic sensitivity. The radiological studies obtained included kidney, ureter, and bladder ultrasound (KUB US), and, if indicated, voiding cysto-urethrogram (VCUG) and dimercapto succinic acid scan (DMSA). *E. coli* strains were identified using the automated VITEK 2 system (BioMérieux, Marcy l’Etoile, France), which employs biochemical testing and pattern recognition for bacterial identification. Also, the VITEK 2 system was utilized to screen for ESBL production in all bacterial strains isolated in this study. Specifically, ESBL detection was performed using the NO45 card (bioMérieux), which consists of six wells containing cefepime (1.0 µg/mL), cefotaxime (0.5 µg/mL), ceftazidime (0.5 µg/mL), cefepime combined with clavulanate (1.0 + 10 µg/mL), cefotaxime combined with clavulanate (0.5 + 4 µg/mL), and ceftazidime combined with clavulanate (0.5 + 4 µg/mL). Bacterial growth was tracked using a spectrophotometric scanner. ESBL production was assessed by comparing the growth patterns of wells containing only these antibiotics to those with added clavulanate, in accordance with the criteria of the Clinical and Laboratory Standard Institute (CLSI) [9]. Antibiotic susceptibility was tested for the following antibiotics: ampicillin, amoxicillin-clavulanate, piperacillin/tazobactam, third-generation cephalosporin (ceftriaxone), amikacin, gentamicin, quinolone (ciprofloxacin), nitrofurantoin, and meropenem trimethoprim/sulfamethoxazole (TMP/SMX). An abnormal VCUG indicated diagnosis of vesicoureteral reflux and/or the posterior urethral valve. An abnormal DMSA scan indicated renal scarring or dysplasia [10].

Data were coded and processed using Statistical Package for the Social Sciences (SPSS) version 28 (IBM Corp., Armonk, NY, USA). For quantitative data, the data were analyzed using the mean, standard deviation, median, and both minimum and maximum values. The frequency (count) and relative frequency (%) were employed for categorical data. A comparison of numerical variables was conducted using the non-parametric Mann–Whitney test. The Chi-square (χ^2^) test was applied for the comparison of categorical data. If the expected frequency fell below 5, an exact test was conducted as an alternative. Statistical significance was determined when *p*-values were below 0.05.

## 3. Results

The study analyzed 242 urine samples obtained from 119 children with *E. coli* UTIs. Of these, 20.7% (n = 50) were ESBL producers. The mean age was 5.3 ± 4.3 years. All samples met the inclusion criteria. The study population revealed female predominance in both the ESBL-producing and non-ESBL groups. When comparing ESBL-producing and non-ESBL-producing *E. coli* UTIs, there was no statistically significant difference in terms of patient age, sex, or clinical presentation. However, the hospital stay was significantly longer for the ESBL group (8.4 ± 1.2 days) compared to the non-ESBL group (4.8 ± 0.8 days) (*p* < 0.001). Notably, the ESBL group demonstrated a higher CRP compared to the non-ESBL group (43.1 ± 73.7 vs. 26.1 ± 55.9, respectively; *p* = 0.032). Though the results were not statistically significant, it is notable that the ESBL group exhibited a higher prevalence of abnormal urine in comparison to the non-ESBL group. Despite the discernible difference in creatinine levels between the two groups, it had no significant clinical impact, as the levels were still within normal limits. Furthermore, there were no significant differences observed in other investigations, such as CBC, radiological abnormalities detected via US, VCUG, or DMSA scan. Hydronephrosis was the most prevalent pathology in both groups, followed by VUR, found in 30% and 14% of the patients, respectively (Table 1).

Regarding potential risk factors for ESBL acquisition, antibiotic administration within the last three months, prophylactic antibiotic use, prior UTI (last 3 months), recurrent UTIs, and underlying co-morbidities (*p* = 0.011, <0.001, 0.025, <0.001, 0.013, respectively) had a significant relationship with increased risk of ESBL *E. coli* UTIs. Generally, neurological co-morbidities were the most common (41.1%), followed by underlying genetic disorders (26.7%) (Table 2).

**Figure 1:**
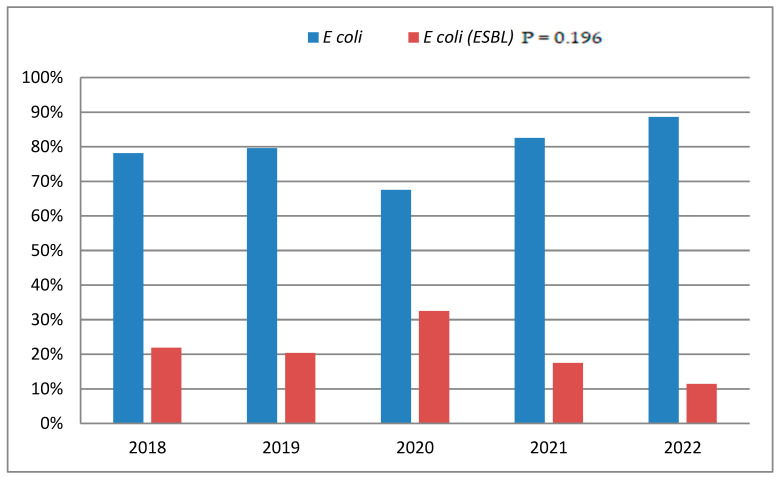
Prevalence of ESBL-producing *E. coli* UTIs (2018–2022).

When assessing the trends related to ESBL *E. coli* UTIs determined based on the year in which the samples were collected, there was no statistically significant change over a five-year period (2018–2022) (*p* = 0.196), but the year 2020 showed the highest ESBL prevalence (32.5%) (Figure 1).

Concerning the antibiotic treatment for ESBL-producing *E. coli* UTIs, third-generation cephalosporin was empirically chosen as the initial antibiotic for 62% (n = 31), followed by aminoglycoside in 22% (n = 11) of these patients, and 16% (n = 8) received meropenem according to a history of admission with an ESBL UTI or a proven culture of an ESBL UTI taken prior to admission. Based on the results of the urine culture and sensitivity, the rates of resistance observed in the ESBL-producing group were 100% for ampicillin and third-generation cephalosporin. Conversely, all ESBL-positive isolates were sensitive to meropenem (Table 3).

## 4. Discussion

ESBL-producing *E. coli* UTIs are a rising concern in public health. The growth of antimicrobial resistance poses a major threat to public health worldwide, especially for ESBL-producing pathogens. For the purpose of directing the proper antibiotic treatment, it is imperative to track and report the prevalence of infections linked to bacteria that produce ESBL [3]. According to a regional research carried out in Saudi Arabia, 33.4% of *E. coli* infections were ESBL producers, demonstrating the severity of this issue within the region [11].

While numerous studies have assessed the occurrence of community-acquired urinary tract infections, similar research in admitted pediatric patients remains scarce, especially in the Middle East, where antibiotic overuse leads to antimicrobial resistance. This study investigated the prevalence, risk factors, and antibiotic resistance of ESBL *E. coli* in hospitalized children with UTIs at a tertiary university hospital.

In the current research of hospitalized children with *E. coli* UTIs over a five-year period, 20.7% were ESBL producers, reflecting the results of a recent local study in Saudi Arabia reporting 18.4% [12]. Other studies reported lower prevalences of ESBL *E. coli* UTIs, 2.6%, 14%, 13%, and 4.5% [3,4,7,13], respectively, which could be due to differences in inclusion and exclusion criteria, as some of these studies included patients with only community-acquired UTIs, and some of them excluded immunocompromised children or those who had recently received antibiotics. Furthermore, our relatively higher percentage may be attributed to several factors, such as the complexity of infections among hospitalized children admitted to this tertiary care institution. Also, some patients were admitted to the hospital due to a prior hospitalization culture of an ESBL UTI. On the other hand, several studies revealed higher rates of ESBL-positive *E. coli* UTIs in different populations, of 26%, 29.5%, 35%, 36%, 47.7%, and 49.1% [14,15,16,17,18,19], respectively. These higher percentages could be attributed to different study populations, distinct timeframes of the studies, and variable inclusion and exclusion criteria. Moreover, overuse of antibiotics by general pediatricians can result in the development of resistant bacteria. Misuse, underuse, or overuse of antibiotics, combined with disregard for local population susceptibility, leads to a rise in multi-drug-resistant ESBL-isolates, potentially contributing to the high prevalence of ESBL UTIs.

In the current study, there was a female predominance, in agreement with previous studies [15,18]. Regarding demographic features, there was no statistically significant difference between the ESBL and non-ESBL *E. coli* UTIs in terms of age or sex, which is in line with other studies [14,17]. In contrast to these findings, one study carried out at a tertiary pediatric hospital in Nepal found that female patients exhibited a greater prevalence of ESBL-producing bacteria (*p* = 0.003) [15], while Alsubaie et al. found that the male sex was identified to be an independent risk factor for ESBL-related urinary tract infections (*p* = 0.026) [12]. 

No notable differences were observed in the clinical presentation between ESBL-producing and non-ESBL *E. coli* UTIs, in agreement with previous studies [7,18], with the exception of a prolonged duration of hospitalization noted in the ESBL cohort (*p* < 0.001), similar to other studies [18,19]. This may be attributable to limited access to oral antibiotics that are effective for treatment of ESBL pathogens and the lag in initiating the proper antibiotic therapy while waiting for urine culture results.

Regarding laboratory parameters, CRP was significantly higher in the ESBL group than in the non-ESBL group, in line with recent research by Alkan et al., who similarly reported higher CRP levels in their ESBL group (*p* = 0.004) [20]. While not statistically significant, it is noteworthy that the ESBL group showed higher prevalence of abnormal urine analysis, notably pyuria and positive nitrites, compared to the non-ESBL group, consistent with a recent local study by Alsubaie et al. [12]. Despite discernible differences in creatinine levels, CBC, and radiological findings, there were no statistically significant differences between the two groups. These findings are in line with the results reported by previous studies [17,19]. Hydronephrosis was as the most prevalent pathology, followed by VUR, found in 30% and 14% of the patients, consistent with a local Saudi study that reported hydronephrosis and VUR as being the most common two urinary tract pathologies, with prevalences of 33% and 18.2%, respectively [12].

The current study found that previous antibiotic use, prophylactic antibiotic use, recurrent UTIs, and underlying co-morbidities significantly increased the risk of ESBL *E. coli* UTIs, consistent with the literature [4,7,18,19,21]. Despite a greater occurrence of genitourinary abnormalities in the ESBL group, the variation was not statistically significant (*p* = 0.095). Several studies have reported that genitourinary abnormalities represent a significant risk factor in ESBL acquisition [4,12,18]. Contrary to these results, a newly conducted study by Qusad et al. revealed that genitourinary abnormalities were less prevalent among their ESBL group compared to the non-ESBL group [17]. These differences could be attributed to different inclusion and exclusion criteria, as well as variability in the numbers of patients who received radiological assessment via US and VCUG.

This study found no significant change in ESBL UTI trends from 2018–2022, but the year 2020 had the highest prevalence, consistent with a recent Saudi study by Altamimi et al. (2024) that showed no statistically significant change during the period from 2018 to 2022. Nevertheless, they revealed an evident surge in ESBL production observed after 2019. These changes in the prevalence around the period of 2019 to 2020 may have been influenced by the COVID-19 pandemic in a number of demographic groups and healthcare settings. The rise in ESBL-producing isolates at the beginning of the COVID-19 pandemic could be attributed to increased antibiotic use as a precautionary measure, altered infection control practices by healthcare providers, and longer hospitalizations, which could increase the risk of hospital-acquired infections, including those caused by ESBL-producing organisms [16]. Another study that investigated Asians and Aboriginal Americans determined that ESBL-producing *E. coli* UTIs fell by an average of 0.22% annually, from 9.1% in 2015 to 8% in 2020 [3].

In terms of antibiotic treatment for ESBL *E. coli* UTIs, third-generation cephalosporin was chosen empirically as the first antibiotic in 62% of the patients, followed by aminoglycoside in 22% and meropenem in 16%. These results support the antibiotics that Albaramki et al. empirically selected, where 55.8%, 19.5%, and 18.2% of the patients receivedthird-generation cephalosporin, aminoglycoside, and carbapenem, respectively [18]. 

Regarding specific culture-based specific, the resistance rates of the ESBL isolates were 100% for ampicillin and third-generation cephalosporin, indicating that these drugs were consistently ineffective against ESBL-producing *E. coli*. Conversely, all ESBL-positive isolates were sensitive to meropenem, indicating its high effectiveness against ESBL-producing *E. coli*. Furthermore, amikacin, as a non-carbapenem alternative, represented as a suitable option for treating pediatric ESBL-UTIs and nitrofurantoin to some extent. The resistance of TMP/SMX was also relatively high, 64%, posing a major problem for the therapeutic approach. Amoxicillin-clavulanate and quinolones showed lower resistance rates compared to ampicillin or a third-generation cephalosporin; however, they still had substantial resistance. Resistance rates to piperacillin/tazobactam and gentamicin were 20% and 22%, respectively, suggesting that these antibiotics may still work as well against ESBL-producing *E. coli*. Consistent with the Saudi study by Altamimi et al. [16], Table 4 illustrates the antimicrobial resistance of the ESBL *E. coli* in the current study in comparison with previous similar studies.

The current study has some limitations. Owing to the retrospective design of the study, complete data were not available for all children, such as missing results of some investigations performed outside our institute. Also, this study was a single-center study carried out in the Western region, so it could not adequately depict the trends of antimicrobial resistance in other parts of Saudi Arabia. Moreover, the relatively small number of isolates available for analysis each year may have limited the statistical power to detect significant trends in antibiotic resistance over time. Additionally, we did not involve children who received treatment in outpatient clinics for UTIs. Therefore, future prospective studies are required to evaluate ESBL UTIs in both inpatient and outpatient settings.

## 5. Conclusions

In conclusion, there is a high prevalence (20.7%) of ESBL production among children hospitalized with *E. coli* UTIs, particularly among those with previous antibiotic use within the last three months, prophylactic antibiotic use, recurrent UTIs, and underlying co-morbidities. Identifying at-risk patients will guide empirical antibiotic therapy while awaiting culture results. Given the 100% resistance to third-generation cephalosporin in ESBL-producing *E. coli*, these antibiotics may not be suitable for empiric therapy in such cases, especially for children with a history of recent use of a third-generation cephalosporin. Alternative empiric therapies, such as amikacin or meropenem, should be considered to ensure effective treatment and reduce the risk of antibiotic resistance. Also, antimicrobial stewardship is crucial to curb the rise in antibiotic resistance. Notably, all ESBL-positive isolates were sensitive to meropenem. Furthermore, amikacin presented as an appropriate non-carbapenem alternative for treating pediatric ESBL UTIs. Proper antibiotic stewardship is essential to avoid complications from inappropriate antibiotic use.

## Figures and Tables

**Table 1:** Subject characteristics of *E. coli* (non-ESBL) vs. ESBL *E. coli* UTI; total samples (n = 242).

Characteristics	*E. coli* (Non-ESBL)	ESBL *E. coli*	*p*-Value
	Mean ± SD or n (%)	Mean ± SD or n (%)	
	Total (n = 192)	Total (n = 50)	
Sex			0.121
Male	62 (32.3%)	22 (44%)	
Female	130 (67.7%)	28 (56%)	
Age: mean ± SD (years)	5.1 ± 4.2	6.1 ± 4.6	0.091
Clinical presentation			
Length of hospital stay (days)	4.8 ± 0.8	8.4 ± 1.2	**<0.001**
Fever	66 (34.4%)	21 (42%)	0.317
Urinary symptoms	58 (30.2%)	21 (42%)	0.113
Urine analysis			
Pyuria	57 (38%)	19 (50%)	0.178
Positive nitrites	62 (41.6%)	18 (48.6%)	0.439
CBC			
WBC (K/µL)	10.2 ± 5.8	9.4 ± 4.9	0.59
HB (g/dl)	11.8 ± 2.8	11.1 ± 2.1	0.231
Platelets (K/µL)	328.6 ± 161.4	346.9 ± 143.3	0.161
CRP (mg/L)	26.1 ± 55.9	43.1 ± 73.7	**0.032**
BUN (mmol/L)	4.7 ± 4.9	6.5 ± 7.5	0.114
Creatinine (µmol/L)	43.2 ± 40.4	59.5 ± 64.3	0.605
Abnormal KUB US	50 (56.8%)	13 (50%)	0.539
Abnormal VUCG	11 (61.1%)	8 (57.1%)	0.821
Abnormal DMSA scan	6 (75%)	1 (20%)	0.103

Bolded values are significant at *p*-value < 0.05. Abbreviations: ESBL *E.coli*, extended-spectrum beta-lactamase-producing *Escherichia coli*; CBC, complete blood count; WBC, white blood cells; HB, hemoglobin; CRP, C-reactive protein; BUN, blood urea nitrogen; KUB, kidney, ureter, and bladder; US, ultrasound; VCUG, voiding cysto-urethrogram; DMSA, dimercapto succinic acid.

**Table 2:** Risk factors associated with the development of ESBL *E. coli* UTIs.

Variables	*E. coli* (Non ESBL) n (%) Total (n = 19)	ESBL *E. coli* n (%) Total (n = 50)	*p*-Value
Prior antibiotic use (last 3 months)	100 (52.1%)	36 (72%)	**0.011**
Prior beta-lactam use (last 3 months)	61 (31.8%)	28 (56%)	**0.002**
Prophylactic antibiotic use	25 (13%)	17 (34%)	**<0.001**
Prior UTI (last 3 months)	85 (44.3%)	31 (62%)	**0.025**
Recurrent UTI	93 (48.4%)	40 (80%)	**<0.001**
Prior hospitalization (last 3 months)	71 (37%)	25 (50%)	0.094
Prior GU surgery (last 3 months)	31 (16.1%)	6 (12%)	0.468
Genitourinary abnormalities	50 (26%)	19 (38%)	0.095
Urinary tract device	10 (5.2%)	2 (4%)	1
Constipation	17 (8.9%)	5 (10%)	0.785
Immunosuppression	21 (10.9%)	4 (8%)	0.543
Co-morbidities	136 (70.8%)	44 (88%)	**0.013**

Bolded values are significant *p*-value < 0.05. Abbreviations: *E. coli*, *Escherichia coli*; ESBL, extended-spectrum beta-lactamase; UTI, urinary tract infection; GU, genitourinary.

**Table 3:** Antibiotic resistance rates (%) of ESBL *E. coli* urine isolates.

Antibiotics	ESBL *E. coli* (Total n = 50) Resistance n (%)
Amoxicillin-clavulanate	20 (40%)
Ampicillin	50 (100%)
Piperacillin/tazobactam	10 (20%)
Third-generation cephalosporin (ceftriaxone)	50 (100%)
Amikacin	1 (2%)
Gentamicin	11 (22%)
Quinolone (ciprofloxacin)	28 (56%)
TMP/SMX	32 (64%)
Nitrofurantoin	4 (8%)
Meropenem	0 (0%)

Abbreviations: *E. coli*, *Escherichia coli*; ESBL, extended-spectrum beta-lactamase; TMP/SMX, trimethoprim/sulfamethoxazole.

**Table 4:** Antibiotic resistance rates (%) of ESBL *E. coli* isolates in the present study and previous studies.

Antibiotic Resistance (%)	Present Study, 2024 (Saudi Arabia)	Albaramki et al., 2019 (Jordan) [18]	Koçak et al., 2016 (Turkey) [19]	Collingwood et al., 2023 (USA) [3]	Vachvanichsanong et al., 2020 (Thailand) [14]	Pantha et al., 2024 (Nepal) [15]	Alsubaie et al., 2023 (Saudi Arabia) [12]	Altamimi et al., 2024 (Saudi Arabia) [16]
Amoxicillin-clavulanate	40	94.8	85.7	-	-	100	40	96
Carbapenem	0 ^a^	1.3 ^n^	0 ^b^	0 ^a^	0 ^a,b^	0 ^b^	0.7 ^a,b^	0 ^a^
Quinolone	56	54.5	37.5	58.6	-	74	65	46
Nitrofurantoin	8	53.2	50	14.8	-	5	32	18
Third-generation cephalosporin	100	98.7	100	-	100	92	-	100
TMP/SMX	64	-	71.4	65.7	68	74	72	67
Piperacillin/tazobactam	20	-	51.8	-	22	-	-	18
Gentamicin	22	54.5	25	36	58	32	21	28
Amikacin	2	32.5	10.7	-	11	-	0.7	1

^a^ meropenem, ^b^ imipenem, ^n^ non-specified carbapenem. Abbreviations: TMP/SMX, trimethoprim/sulfamethoxazole.

## Data Availability

The original contributions presented in this study are included in the article; further inquiries can be directed to the corresponding author.
